# Use of Research in the Nursing Practice: from Statistical Significance to Clinical Significance

**DOI:** 10.17533/udea.iee.v41n3e12

**Published:** 2023-10-31

**Authors:** R. Mauricio Barría P.

**Affiliations:** 1 RN, M.Sc, Ph.D. Director of the Institute of Nursing, Faculty of Medicine, Universidad Austral de Chile. email: rbarria@uach.cl Universidad Austral de Chile Faculty of Medicine Universidad Austral de Chile Chile rbarria@uach.cl

**Keywords:** nursing research, data interpretation, statistical, clinical relevance, nursing, practical, evidence-based practice, investigación en enfermería, interpretación estadística de datos, relevancia clínica, enfermería práctica, práctica clínica basada en la evidencia, pesquisa em enfermagem, interpretação estatística de dados, relevância clínica, enfermagem prática, prática clínica baseada em evidências

## Abstract

**Objective.:**

Within the context of evidence-based practice, this article exposes the reflection on the understanding and usefulness of the information provided by the research findings shared in reports and research publications, exposing differences based on the interpretation of statistical significance and clinical significance.

**Content synthesis.:**

Basic aspects of the meaning and use of the information reported by research on p value (statistical significance) and the value and usefulness of these results are analyzed and exemplified, contrasting the value for the practice of an additional judgment on clinical significance. In addition to establishing conceptual differences, the need is highlighted for nurses to have the competencies to differentiate and apply each of them according to the clinical contexts of their potential implementation.

**Conclusion.:**

The real usefulness of research about interventions within the context of nursing care is given by its real application and reach for the practice and benefit for patients. For this to occur, nurses must interpret adequately the information provided by scientific publications and other research reports.

## Resumen

**Objetivo.:**

En el contexto de una práctica basada en evidencia, este artículo expone la reflexión sobre la comprensión y utilidad de la información que proveen los hallazgos de investigación reportados en informes y publicaciones de investigación, exponiendo las diferencias a partir de la interpretación de la significancia estadística y significancia clínica.

**Síntesis del contenido.:**

Se analizan y ejemplifican aspectos básicos sobre el significado y uso de la información que reportan las investigaciones sobre valor p (significancia estadística) y el valor y utilidad de estos resultados contrastando el valor para la práctica de un juicio adicional sobre significancia clínica. Además de establecer diferencias conceptuales, se resalta la necesidad de que las enfermeras tengan las competencias para diferenciar y aplicar cada uno de ellos según los contextos clínicos de su potencial implementación.

**Conclusión.:**

La real utilidad de la investigación sobre intervenciones en el contexto del cuidado de enfermería está dada por su real aplicación y alcance para la práctica y el beneficio para los pacientes. Para que ello ocurra, las enfermeras deben interpretar adecuadamente la información que proveen las publicaciones científicas y otros reportes de investigación.

## Resumo

**Objetivo.:**

No contexto de uma prática baseada em evidências, este artigo apresenta a reflexão sobre a compreensão e utilidade da informação fornecida pelos resultados da investigação relatados em relatórios de investigação e publicações, expondo as diferenças com base na interpretação da significância estatística e da significância clínica.

**Síntese de conteúdo.:**

Aspectos básicos sobre o significado e uso das informações relatadas pelas pesquisas sobre valor p (significância estatística) e o valor e utilidade desses resultados são analisados ​​e exemplificados, contrastando o valor para a prática de um julgamento adicional sobre significância clínica. Além de estabelecer diferenças conceituais, destaca-se a necessidade de o enfermeiro ter competências para diferenciar e aplicar cada uma delas de acordo com os contextos clínicos de seu potencial implementação.

**Conclusão.:**

A real utilidade da investigação sobre intervenções no contexto dos cuidados de enfermagem é dada pela sua real aplicação e âmbito de prática e benefício para os pacientes. Para que isso ocorra, os enfermeiros devem interpretar adequadamente as informações fornecidas pelas publicações científicas e outros relatórios de pesquisa.

## Introduction

Daily, nurses face dilemmas in clinical practice to make decisions about caring for patients, a situation in which research contributes to the scientific rigor of daily practice, allowing improvements when applying knowledge in favor of caring for patients.^1^ Thus, the use of research in nursing practice is fundamental to provide quality and evidence-based care. Nursing research began with Nightingale when she investigated the morbidity and mortality of patients during the Crimean War. From there, it was again taken up until the 1930s and 1940s when nurses began to conduct studies on nursing education. During the 1950s and 1960s, nurses and nursing roles were the focus of research, until the end of the 1970s and 1980s, the aim of research centered on studies to improve the nursing practice. In the 1990s, research sought to describe nursing phenomena, test the effectiveness of nursing interventions, and examine the results on patients. Currently, nursing research of the 21^st^ century considers quality studies through the use of a variety of methodologies, synthesis of research findings, use of this evidence to guide the practice and examine the results of the evidence-based practice.^2^

A key aspect of using research in the nursing practice is the application of scientific evidence on clinical decision making. When basing decisions on the best evidence available, nurses can provide more effective and safe care, but this requires reviewing and critically evaluating published studies, considering their validity, relevance and applicability to the specific clinical situation. It is within this context that it is proven that sufficient knowledge is still not available on the part of clinicians to adequately evaluate the research findings to be translated into practice. Examples of this are the assessments of the concepts of statistical significance and clinical significance. In the nursing field, clinical significance and statistical significance are fundamental concepts in evidence-based decision making. These concepts permit evaluating the relevance of research results and their application in the clinical practice. Although both terms are related, it is important to comprehend their differences and how they complement each other to provide quality care to patients.

In quantitative research, nurse researchers are expected to assess, understand, and report the results of their studies using appropriate statistical methods, as well as provide a description of the clinical relevance of their findings to make sure an article is not just a description of new knowledge, but that it is useful for evidence-based practice. A focus on the magnitude of the effects, rather than simply their statistical significance (p value), could provide the opportunity to link data generated in each study with the clinical relevance these could provide. Reaching this statistical comprehension in the nursing practice will improve directly or indirectly the research articles and will facilitate communication between statisticians and clinical professionals to improve the reporting of research and disseminating findings. However, it is difficult to expect for all nurses to be experts in statistics and, additionally, to ensure that statisticians have the vision and clinical knowledge, so a dialogue must be achieved between both visions.^3^

It is common to read research reports (publications) or see presentations of scientific sessions and conferences in which researchers, when reporting on comparisons of therapeutic or preventive interventions, use the expressions "statistical significance" or "statistically significant". This entails the danger of confusing clinical and statistical significance. Although, traditionally, reports of research results focus heavily on statistical significance, numerous errors have been noted when using this as the only approach to interpret and apply research findings. Furthermore, some warn that decisions should never be made based only on a significance test or p value and that in reality p values continue to be poorly understood and widely misused.^4^ In the expression of statistical analyses, undoubtedly, the most universally recognized is the p value. Most people have the notion that a p value < 0.05 means a statistically significant difference among groups being compared. However, the traditional interpretation of statistical significance as *p* < 0.05 is arbitrary and errors have been observed in its interpretation, besides, it is expected that they will change according to the sample size, observing that bigger samples provide results with smaller p values.^5^ This article sought to provide basic and conceptual information about the implications of the terms statistical significance and clinical significance and make people reflect on the understanding and usefulness of the information provided by research findings shared in reports and research publications.

## Statistical significance

Overall, it could be understood that statistical significance is a term indicating that the results obtained in an analysis of data from a sample are unlikely to be due to chance at some specific level of probability, given the veracity of a null hypothesis. Thus, a p value represents the probability of calculating a statistical test from the data from a sample (*e.g*., a mean difference between two groups) that is equal to or more extreme than that observed in the sample data assuming that the null hypothesis is actually true. In other words, the p value measures how compatible the data from the sample is with the null hypothesis (*e.g*., there are no differences between the groups).^6^^-^^8^

Significance tests have become an integral part of the process of quantitative research in scientific disciplines, including nursing. These tests complement the scientific method and offer an objective dimension in the analysis of studies to answer questions from the practice. Studies use a predefined threshold to determine when a p value is small enough to support a hypothesis in the study. Conventionally, this threshold is set at a p value of 0.05, equivalent to a type-I error probability level (alpha level or *p*) of 5% and whose determination is achieved through hypothesis tests. However, there may be situations and justifications for studies to use a different threshold, if appropriate. 

As outlined, researchers typically develop two types of hypotheses, a null hypothesis (H_0_) and an alternative hypothesis (H_1_). The null hypothesis establishes that no relation exists (or there is no difference) among groups in the study of variables of interest and any relationship that can be observed is due to chance or sampling fluctuations. The alternative hypothesis affirms that a relation or difference exists, which is not due to chance and is assumed real (example in Table 1). 

**Table 1:** Example of an intervention study

Nurse researchers propose a study within the context of neonatal care in which they expect to evaluate if an effect exists on the abandonment of breastfeeding from an intervention denominated “Breastfeeding Support Program (BSP)”. For this, they assign randomly mothers of children hospitalized in the neonatal unit to an experimental group, which receives the individualized breastfeeding support program, while other mothers were assigned to the control group, which receives standard or habitual care and education. Within this scenario, the researchers would hypothesize that a difference exists in the proportion of mothers who abandon breastfeeding one month after hospital discharge depending on whether or not they receive the intervention, which is denominated research, working or alternative hypothesis (H_1_). Moreover, and given that there is always the possibility of no difference among the groups, a hypothesis must also be established that reflects this lack of difference (effect), denominated null hypothesis (H_0_); that is, not finding differences in the proportion of abandonment of breastfeeding among the groups upon ending the monitoring.

It should be mentioned that, in studies using a sufficiently large sample, a statistical test almost always will demonstrate a significant difference, unless there is no effect at all, that is, when the effect size is exactly zero. Furthermore, very small differences, even being significant, often make no sense and do not provide value or utility for the practice. Therefore, reporting only the significant p value for an analysis is not adequate for readers to fully understand the results.^8^ As reinforced by Polit,^9^ an important reason for not homologating statistical significance with clinical significance is precisely because statistical significance is strongly affected by sample size and, thus, in a study with a large sample, the statistical power is high and the risk of committing a type-I error (erroneously concluding that no relationship exists among the variables) is low. Polit exemplifies it thus, “…with a sample size of 500, a modest correlation of r = 0.10 is statistically significant at *p* < 0.05, even though such a weak relationship may have little practical importance”.^(9, p.18)^

## Clinical significance

Given that no universal agreement exists on the definition of clinical significance, various approaches exist for its evaluation. In addition, it has not received sufficient attention in the specific nursing literature reflecting that recent progress in measuring the clinical importance have not penetrated to a large extent in nursing.^9^ It is even described that its use has been carried out inconsistently and without always considering a measurable result for the patient.^10^ Overall, clinical significance refers to the practical importance of a result in real life or the benefits of research results for users and patients. It often measures the magnitude of the relation between an independent variable and an outcome variable. As expressed, conceptually, the importance of clinical significance is illustrated in its comparison with statistical significance. This is that, while the p values of a statistically significant finding indicate the probability that a change is caused by chance, clinical significance establishes whether this change or difference is large enough to have implications in practice. As anticipated, it is recognized that a p value cannot express the clinical relevance or importance of the effects observed from an intervention and specifically, does not provide details on the magnitude of an effect. So, although a p value is significant (conventionally < 0.05), it is possible that the difference between groups is small. This phenomenon is especially common with larger samples in which comparisons can yield as a result statistically significant differences that are actually not clinically significant.^10^

As proposed by Bruner *et al.*,^10^ numerous problems exist associated with using clinical significance in the nursing literature. Among these, they highlight the lack of consensus on the use of the term from a multiplicity of opinions, definitions, and uses. In turn, given that clinical significance is commonly based on the researcher’s judgement, the term is sometimes used subjectively and the findings are prone to bias in favor of positive results. Lastly, most studies do not incorporate the patient’s perspective. Thus, it is necessary to highlight that besides this vision from the clinician’s perspective, there are proposals that have been gaining space in the assessment of research and its applicability in the practice and which is guided from the very patient’s perspective, such as the concept of minimal clinically important difference.^11^

## Application of statistical significance and clinical significance

To illustrate the relation between statistical and clinical significance, let us consider the fictitious scenario in which a group of research nurses studies a breastfeeding support program to reduce early abandonment of breastfeeding after hospital discharge (Table 1). Supposing that the result or outcome is measured in a binary scale, like maintains/abandons breastfeeding, at the end of the study, a significant difference is reported on the proportion of abandonment of breastfeeding between both groups. Although this result indicates that the difference between the study groups is probably not due to chance, it only provides partial information, given that, strictly speaking, statistical significance has not proven anything. When a result is deemed statistically significant, it is understood that an independent variable has an effect upon a dependent variable but does not prove that something will occur, given that the p value does not express magnitude. 

**Figure 1:**
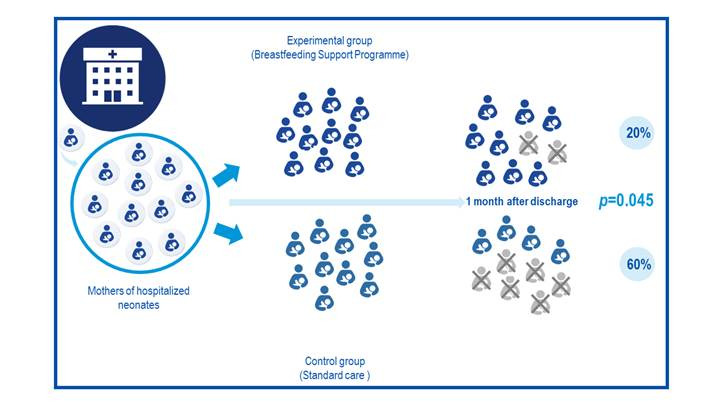
Example 1 of effect of a breastfeeding intervention, differences between groups and p value

It is necessary to know whether or not this finding, in addition to being a statistically significant difference, has any clinical value. Reviewing the results, it is confirmed that abandonment of breastfeeding one month after hospital discharge in the experimental group was 20%, while in the control group it was 60% (Figure 1). This drastic reduction in abandonment of breastfeeding in the experimental group could be considered relevant given the known benefits of breastmilk in different settings, both for the mother and child, which supposes that the potential population benefitted would justify implementing a program within the hospital context, like the one studied. Additionally, the researchers have reported a *p* value = 0.045, which under the conventional assumption of the limit value assigned to it of 5% (0.05), also corroborates statistical significance. 

**Figure 2:**
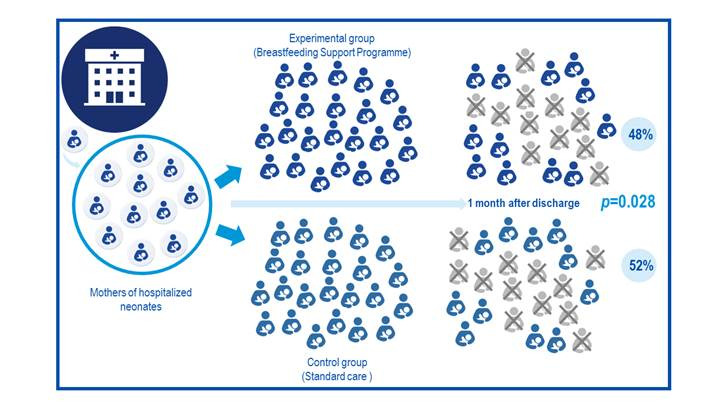
Example 2 of effect of a breastfeeding intervention, differences between groups and p value.

Now, let's suppose another scenario where in a similar proposal researchers recruited more participants for their research, obtaining a result that highlighted that in the experimental group abandonment of breastfeeding one month after discharge was 48% while in the control group it was 52%. Although the statistical significance reported by the researchers, given the p value = 0.028, indicates statistically significant differences between the groups, it is necessary to consider whether the merely 4% reduction in the outcome studied justifies implementing an individualized breastfeeding support program. Thereby, researchers and readers of the research report will have evidence to discuss carefully this statistically significant finding, highlighting the apparently marginal clinical importance of the resources required to implement the intervention. Further, in comparing the examples mentioned, differences in p values obtained are expressed given the influence of the also different sample sizes. In this case, it is noted that although the p value from the example in Figure 2 is lower than that in Figure 1 (0.028 Vs. 0.045), clarifying that a smaller p value does not necessarily guarantee clinical significance. 

Hence, as reflected in this example, the clinical significance of the research results is best evaluated by making a judgment based on clinical experience, assessing the benefits, costs, and risks associated with the findings of each study. If the benefits (or effects) reported clearly outweigh the risks and the effect is large enough, then a statistically significant finding is also clinically significant.

To end, it is worth mentioning that besides a purely qualitative view of how large or small a difference or effect is found in the results of a study, the size of the effect is estimated with different indices. Overall, a difference exists between those analyzing effect sizes between groups and those analyzing measures of association between variables. For two independent groups, the size of the effect can be measured through the standardized difference between to measurements. Cohen’s d term is an index of the size of the effect and classifies it into small (d=0.2), medium (d=0.5), and large (d=0.8) effect sizes.^8^ Readers should delve into this and other concepts of effect size measurements.

## Conclusion

This article has sought to reflect on the need for clinical nurses, as well as those who use research findings for their potential application, to expand the evaluation of study results beyond the merely statistical evaluation and contrast this information with clinical usefulness and its impact on patients and population. It is clear that exclusive dependence on statistical significance to assign meaning and importance to research findings continues being a problem in different areas of health sciences and in nursing. Upon contrasting conceptually, the scope of the terms statistical significance and clinical significance, it is expected that evidence-based decisions will be made cautiously, understanding that statistical significance allows inferences to be made about the results of a study, but this is not sufficient to make sound recommendations about the potential clinical benefits from those findings. Consequently, researchers and clinicians need to always assess the clinical importance of the research findings and weigh statistically significant results within the context of their importance for the practice and benefit in patients.

## References

[1] 1. Barría RM. Nursing Research, Dissemination of Knowledge and its Potential Contribution to the Practice. Invest Educ Enferm. 2022; 40(3):e01. 10.17533/udea.iee.v40n3e01 PMC10017141 36867774

[2] 2. Gray JR. Evolution of research in building evidence-based nursing practice. In: Gray JR, Grove SK, editors. Burns & Grove’s The Practice of Nursing Research: Appraisal, Synthesis, and Generation of Evidence, 9th Edition. Elsevier. 2020.

[3] 3. Visentin DC, Hunt GE. What do the stats mean? Improving reporting of quantitative nursing research. Int. J. Ment. Health Nurs. 2017; 26(4):311-3. 10.1111/inm.12352 28670792

[4] 4. Hayat MJ, Staggs VS, Schwartz TA, Higgins M, Azuero A, Budhathoki C, et al. Moving nursing beyond p < .05. Nurs. Outlook. 2019; 67(5):509-10. 10.1016/j.outlook.2019.06.010 31375344

[5] 5. Staggs VS. Pervasive errors in hypothesis testing: Toward better statistical practice in nursing research. Int. J. Nurs. Stud. 2019; 98:87-93. 10.1016/j.ijnurstu.2019.06.012 31349121

[6] 6. Phillips MR, Wykoff CC, Thabane L, Bhandari M, Chaudhary V. Retina Evidence Trials InterNational Alliance (R.E.T.I.N.A.) Study Group. The clinician's guide to p values, confidence intervals, and magnitude of effects. Eye (Lond). 2022; 36(2):341-2. 10.1038/s41433-021-01863-w PMC8807597 34837035

[7] 7. Mueller M. Using P Values, Why and How? J. Wound Ostomy Continence Nurs. 2020; 47(5):521-2. 10.1097/WON.0000000000000686 32970038

[8] 8. Sullivan GM, Feinn R. Using Effect Size-or Why the P Value Is Not Enough. J. Grad. Med. Educ. 2012; 4(3):279-82. 10.4300/JGME-D-12-00156.1 PMC3444174 23997866

[9] 9. Polit DF. Clinical significance in nursing research: A discussion and descriptive analysis. Int. J. Nurs. Stud . 2017; 73:17-23. 10.1016/j.ijnurstu.2017.05.002 28527824

[10] 10. Bruner S, Corbett C, Gates B, Dupler A. Clinical significance as it relates to evidence-based practice. Int. J. Nurs. Knowl. 2012; 23(2):62-74. 10.1111/j.2047-3095.2012.01205.x 23281882

[11] 11. Monrroy Uarac M, Barría Pailaquilén M. Diferencia Mínima Clínicamente Importante: Centrando la Toma de Decisiones en el Paciente. Kinesiología. 2022; 41(3):300-4.

